# Isolation of RNA from Acute Ischemic Stroke Clots Retrieved by Mechanical Thrombectomy

**DOI:** 10.3390/genes12101617

**Published:** 2021-10-14

**Authors:** Vincent M. Tutino, Sarah Fricano, Kirsten Frauens, Tatsat R. Patel, Andre Monteiro, Hamid H. Rai, Muhammad Waqas, Lee Chaves, Kerry E. Poppenberg, Adnan H. Siddiqui

**Affiliations:** 1 Canon Stroke and Vascular Research Center, University at Buffalo, Buffalo, NY 14203, USA; sefrican@buffalo.edu (S.F.); kfrauens@buffalo.edu (K.F.); tatsatra@buffalo.edu (T.R.P.); amonteiro@ubns.com (A.M.); hrai@ubns.com (H.H.R.); mwaqas@ubns.com (M.W.); leechave@buffalo.edu (L.C.); kerrypop@buffalo.edu (K.E.P.); asiddiqui@ubns.com (A.H.S.); 2 Department of Pathology and Anatomical Sciences, University at Buffalo, Buffalo, NY 14260, USA; 3 Department of Neurosurgery, University at Buffalo, Buffalo, NY 14260, USA; 4 Department of Mechanical & Aerospace Engineering, University at Buffalo, Buffalo, NY 14260, USA; 5 Department of Radiology, University at Buffalo, Buffalo, NY 14260, USA

**Keywords:** acute ischemic stroke, large vessel occlusion, RNA extraction, method, RNA sequencing, quantitative polymerase chain reaction

## Abstract

Mechanical thrombectomy (MT) for large vessel acute ischemic stroke (AIS) has enabled biologic analyses of resected clots. While clot histology has been well-studied, little is known about gene expression within the tissue, which could shed light on stroke pathophysiology. In this methodological study, we develop a pipeline for obtaining useful RNA from AIS clots. A total of 73 clot samples retrieved by MT were collected and stored in RNALater and in 10% phosphate-buffered formalin. RNA was extracted from all samples using a modified Chemagen magnetic bead extraction protocol on the PerkinElmer Chemagic 360. RNA was interrogated by UV–Vis absorption and electrophoretic quality control analysis. All samples with sufficient volume underwent traditional qPCR analysis and samples with sufficient RNA quality were subjected to next-generation RNA sequencing on the Illumina NovaSeq platform. Whole blood RNA samples from three patients were used as controls, and H&E-stained histological sections of the clots were used to assess clot cellular makeup. Isolated mRNA was eluted into a volume of 140 µL and had a concentration ranging from 0.01 ng/µL to 46 ng/µL. Most mRNA samples were partially degraded, with RNA integrity numbers ranging from 0 to 9.5. The majority of samples (71/73) underwent qPCR analysis, which showed linear relationships between the expression of three housekeeping genes (*GAPDH*, *GPI*, and *HPRT1*) across all samples. Of these, 48 samples were used for RNA sequencing, which had moderate quality based on MultiQC evaluation (on average, ~35 M reads were sequenced). Analysis of clot histology showed that more acellular samples yielded RNA of lower quantity and quality. We obtained useful mRNA from AIS clot samples stored in RNALater. qPCR analysis could be performed in almost all cases, while sequencing data could only be performed in approximately two-thirds of the samples. Acellular clots tended to have lower RNA quantity and quality.

## 1. Introduction

Acute ischemic stroke (AIS) is the fifth leading cause of death worldwide. In the U.S., about 7 million people over the age of 20 have had a stroke, and every year approximately 800,000 Americans will experience a stroke, causing 130,000 deaths and persistent neurological deficits in many survivors. The use of mechanical thrombectomy (MT) in the treatment of AIS is now mainstream therapy for large vessel occlusions. The advent of this technology has enabled the biologic investigation of resected clot material. Histological analyses have demonstrated clot composition could be used to inform clinical management of AIS patients. For example, the fibrin/platelet (FP), red blood cell (RBC), and white blood cell (WBC) content of the clot have been associated with AIS etiology [1,2,3,4], and thus could be used in diagnosing cryptogenic cases. Yet, outside of basic histologic analyses, little is known about the biology of these tissue samples, which may provide additional information into stroke pathophysiology and may open the door to more effective diagnostics from clot analysis.

Transcriptome profiling is a powerful tool that reveals RNA expression patterns that define cellular responses associated with biologic processes and disease states. These data are valuable in both understanding biologic and pathobiologic cellular mechanisms and developing useful biomarkers. Hence, clot mRNA could be extremely useful for such purposes. However, since retrieved clot samples are often small and may be acellular (in the case of fibrin-rich clots), isolating high volumes of high-quality RNA may be difficult. To our knowledge, only two reports in the published literature have demonstrated analysis of RNAs extracted from retrieved AIS clot samples [5,6]. However, these studies did not adequately detail extensive downstream applications, such as RT-qPCR analysis or RNA sequencing, which are crucial tools for understanding the transcriptomes of AIS clots.

Herein, we sought to demonstrate an RNA isolation pipeline that we tailored to the PerkinElmer Chemagic 360 platform. Combining RNALater-based collection/storage, manual sample disruption, and Chemagen magnetic bead extraction, RNA was isolated for use in downstream applications. We performed both traditional qPCR and next-generation RNA sequencing on clot RNA and RNA isolated from peripheral whole blood, as a control. Furthermore, we investigated the histology of the clots to determine if RNA quality was affected by clot composition. We hope this report provides a useful pipeline that can enable further investigations of RNA expression in clot samples retrieved from large vessel AIS cases.

## 2. Materials and Methods

### 2.1. Patient Recruitment and Sample Collection

This study was approved by the University at Buffalo Institutional Review Board (STUDY00002092). All methods were carried out in accordance with the approved protocol and written informed consent was obtained from all subjects. We included patients in this study if they were receiving MT procedures for the treatment of AIS and had sufficient clot material collected. Patients without usable clot tissue samples were excluded.

From 9/2018 and 11/2020, clot samples were collected from patients receiving MT by either stent retriever, aspiration, or combined therapy at the Gates Vascular Institute in Buffalo, NY. Immediately after the procedure, participating clinicians placed portions of the retrieved clot material into production-sterile FluidX tubes (Brooks Life Sciences, New York, NY, USA). One FluidX tube was filled with RNALater RNALater (ThermoFisher, Waltham, MA, USA), a storage reagent that quickly penetrates tissue samples to stabilize and protect RNA and inactivate RNases. Another FluidX tube was filled with 10% phosphate-buffered formalin to facilitate downstream histological analyses. After 24 h at room temperature, samples in RNALater were stored at 4 °C for 24 h, then transferred to −80 °C until further processing. Samples in formalin were allowed to fix at room temperature for 24–48 h then transferred into 70% ethanol until further analysis. For a subset of patients, arterial whole blood was collected from the access sheath during the procedure and placed into one 2.5 mL PAXgene Blood RNA tube (PreAnalytiX, Hombrechtikon, Switzerland). After 24 h at room temperature, these samples were stored at −20 °C for 24 h, then transferred to −80 °C until further processing.

### 2.2. RNA Isolation

For RNA isolation, the clot material (typically ranging from 1 mm^3^ to 10 mm^3^) was removed from the RNALater and placed directly into lysis buffer, followed by manual disruption via shearing for ~5 min. Disrupted samples were then transferred to a 24-well plate, incubated in lysis buffer with proteinase K, then processed on the high-throughput PerkinElmer Chemagic 360 instrument that uses Chemagen Technology, as described by the manufacturer (Perkin Elmer, Waltham, MA, USA). In brief, RNA was captured through highly specific binding to DNAseI-treated M-PVA magnetic beads that are attracted to transiently magnetized metal rods. To avoid contamination in the procedure, the rods brought the RNA-bound beads through a series of process solutions (including DNAseI treatment), serial wash buffers, and elution buffers that ultimately yield purified RNA. Whole blood RNA was processed using a complementary protocol and extracted directly from 500 µL aliquots of PAXgene blood RNA tubes. For both clot and whole blood, RNA was eluted in a final volume of 140 µL.

### 2.3. RNA Quantification

The purity and concentration of each RNA sample was measured by absorbance at 260 nm and 280 nm on the Big Lunatic (Unchained Labs, Pleasanton, CA, USA) or a NanoDrop 2000 spectrophotometer (Thermo Scientific, Waltham, MA, USA). A ~50 µL aliquot was reserved for sequencing. For library preparation, RNA concentration was first more precisely measured via the Qbit Assay (Invitrogen, Carlsbad, CA, USA) with a TBS-380 Fluorometer (Promega, Madison, WI, USA). The quality of the RNA samples was measured on an Agilent 2400 Tape Station or an Agilent 2100 BioAnalyzer (Agilent, Las Vegas, NV, USA). RNA samples to be sequenced needed to have acceptable quality, as measured by the RNA integrity number (RIN > 3.0). In the case of unreadable and lower RIN (<3.0), samples were still sequenced if inspection of the electropherogram showed rRNA peaks with minimal gDNA contamination (the average RIN of sequenced RNA was 3.1).

### 2.4. RT-qPCR

We performed a quantitative reverse transcription-polymerase chain reaction (RT-qPCR) as previously described [7,8,9]. Primers were created for three housekeeping genes: *GAPDH, GPI,* and *HPRT1*. For each gene, oligonucleotide primers with an ~60 °C melting temperature and a length of 15–25 nucleotides were designed to produce PCR products with lengths of 50–250 base pairs via Primer3 software and Primer-BLAST (NCBI, Bethesda, MD, USA). The replication efficiency of each primer had been tested by qPCR on serial dilutions of pooled cDNA (primer sequences, annealing temperatures, efficiencies, and product lengths are shown in Supplemental Table S1).

For reverse transcription, cDNA was generated from total RNA using SuperScript III Reverse Transcriptase Kit (ThermoFisher, Waltham, MA, USA) according to the manufacturer’s instructions. Quantitative PCR was run with 10 ng of cDNA in 25 μL reactions in duplicate in the Bio-Rad CFX Connect system (Bio-Rad, Hercules, CA, USA) using the qScript One-Step SYBR Green Master Mix Kit (Quantabio, Beverly, MA, USA), and gene-specific primers at a concentration of 0.02 μM each. The temperature profile consisted of an initial step of 95 °C for 1 min, followed by 40 cycles of 95 °C for 15 s and 60 °C for 1 min, and then a final melting curve analysis from 60 °C to 95 °C for 20 min. Gene-specific amplification for these primers had been demonstrated by a single peak using the Bio-Rad dissociation melt curve. The Ct values for each housekeeping gene were recorded for all samples.

### 2.5. RNA Sequencing

For RNA sequencing, we used the Illumina TruSeq RNA Library Preparation Kit (Illumina, San Diego, CA, USA) that employed a poly-A pull-down for mRNA isolation. Library preps were assessed by the Agilent 2100 BioAnalyzer RNA 6000 Pico Chip (Agilent, Las Vegas, NV, USA). RNA libraries were then subjected to 100-cycle, paired-end sequencing in a NovaSeq6000 system (Illumina, San Diego, CA, USA) and demultiplexed using Bcl2Fastq. Per-cycle base-call (BCL) files generated by the Illumina NovaSeq6000 were converted to per-read FASTQ files using bcl2fastq version 2.20.0.422 using default parameters. The quality of the sequencing was reviewed using FastQC version 0.11.5. Detection of potential contamination was done using FastQ Screen version 0.11.1 [10]. FastQC and FastQ Screen quality reports were summarized using MultiQC version 1.5 [11]. Genomic alignments were performed using HISAT2 version 2.1.0 using default parameters. NCBI reference GRCh38 was used for the reference genome and gene annotation set. Sequence alignments were compressed and sorted into binary alignment map (BAM) files using samtools version 1.3. Counting of mapped reads for genomic features was performed using Subread featureCounts version 1.6.2 using the parameters -s 2 –g gene_id –t exon –Q 60, the annotation file specified with –a was the NCBI GRCh38 reference from Illumina iGenomes. Aggregate quality control data (i.e., alignment statistics and feature assignment statistics) were summarized in MultiQC [11].

To analyze the quality of the RNA sequencing data, we performed several analyses in R. We used DESeq2 version 1.32.0 [12] (including transcripts with 50% expression across all samples and incorporating clot and blood sources in the design matrix) to graph the dispersion of the data using a local fit and to calculate the Cook’s Distance to identify outliers. We also utilized degCheckFactors in DEGreport version 1.28.0 to determine if the median ratio normalization used by DESeq2 is the best size factor to represent depth (distribution of the ratios should be normal). Lastly, we plotted boxplots of log-transformed counts per million (CPM) expression data to determine if any samples had significantly lower or higher expression levels.

### 2.6. Histological Analysis

For histology, formalin-fixed clots (stored in 70% ethanol) were embedded in paraffin blocks, and sectioned at 4 µm on a microtome. Sections were stained with Hematoxylin and Eosin (H&E) stain, as described previously [13]. The stained slides were then imaged by whole slide scanning at 63 X magnification on the ScanScope digital histology platform, Aperio ScansScope AT Turbo (Leica Biosystems, Wetzlar, Germany). Next, quantification of clot composition was performed on digital histology images using the opensource software, Orbit Image Analysis (www.orbit.bio, accessed on 15 January 2021) under the default settings, as described elsewhere [14]. Prior to this analysis, digital slide images were down-sampled to be at the same resolution and resized to be on the same scale. Errors in histology, i.e., out of focus regions, folds, or debris, were digitally removed. Orbit Image Analysis software then trained computational algorithms for image segmentation, classification, and quantification using a support vector machine on a per-sample basis. This pipeline was used to define and annotate RBC, FP, and WBC regions, and calculate their percentages for each slide.

## 3. Results

### 3.1. Study Population

A total of 73 AIS patients (43% male and 57% female, average age of 70 ± 1.7) treated by mechanical thrombectomy met the inclusion criteria for this study (Table 1). The clinical data of the patients are summarized in Table 1. The majority of all cases had an MCA or ICA occlusion (62% and 28%, respectively). For first-pass therapy, stent retrievers were used in 10% of patients, aspiration catheters were used in 14% of patients, and Solumbra was used in 76% of patients. A total of 36% of the patients were treated with IV-tPA prior to MT, whereas 0% received IA-tPA during thrombectomy. The average number of passes for complete revascularization was 1.9 ± 0.15 and, on average, clots were retrieved on pass 1.7 ± 0.15. Whole blood samples were collected from a subset of three patients as control tissue (see Table 1).

### 3.2. Quantity and Quality of AIS Clot RNA

**Figure 1:**
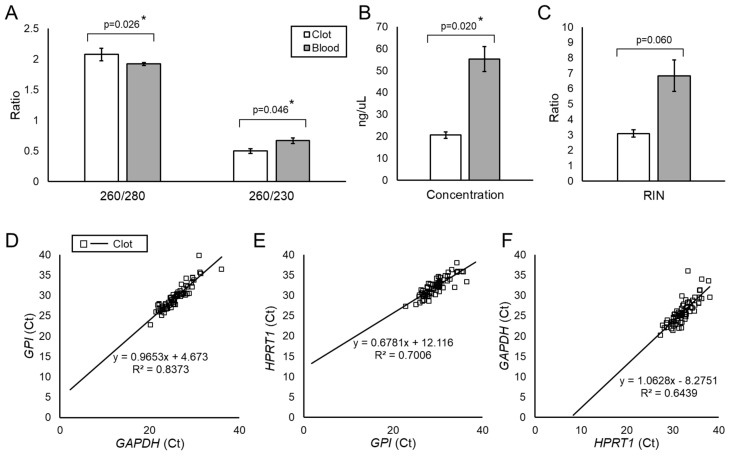
RNA Quality and qPCR Analysis. (**A**)**.** Average 260/280 and 260/230 ratios for clot RNA and whole blood extracted on the PerkinElmer Chemagic 360. Both sample types show 260/280 close to 2.0 (indicating RNA), but lower 260/230 ratios, indicating potential carryover of reagents from processing. (**B**)**.** Blood RNA showed significantly higher average concentrations than clot RNA. (**C**). Blood RNA also had significantly higher average RIN values than clot RNA. (**D**). Ct values from *GAPDH* and *GPI* amplification plotted against each other for each clot RNA sample show a correlation on linear regression analysis (R^2^ = 0.84). A similar trend was present for (**E**). *HPRT1* vs. *GPI* (R^2^ = 0.70) and (**F**). *GAPDH* vs. *HPRT1* (R^2^ = 0.64). Error bars represent standard error. “*” indicates statistical significance, *p* < 0.05 from Student’s *t*-test. (Abbreviations: qPCR = quantitative polymerase chain reaction; RIN = RNA integrity number).

Table 2 shows the RNA quality metrics reported for all clot and blood samples. Clot RNA demonstrated average 260/280 and 260/230 ratios of 2.08 and 0.50, respectively, while whole blood RNA had average 260/280 and 260/230 ratios of 1.92 and 0.67, respectively (Figure 1A). On average, our isolation method yielded 140 µL of clot RNA with a concentration of 26.5 ng/µL (range = 0.80–160 ng/µL) and whole blood RNA with a concentration of 38.3 ng/µL (range = 31.0–45.0 ng/µL) (Figure 1B). Whole blood RNA had a significantly greater concentration than clot RNA, (*p* = 0.020, Student’s *t*-test). Bioanalyzer analysis showed that clot RNA had an average RIN of 3.1 (range = 0–9.5) and whole blood RNA had an average RIN of 6.8 (range = 4.8–8.1). Based on the concentration, the RIN, and assessment of the Bioanalyzer traces to rule out genomic DNA contamination, we selected 48 (66%) clot RNA samples and all three whole blood samples for RNA sequencing analysis.

### 3.3. Most Samples were Amenable to qPCR Analysis

To determine clot RNA viability in downstream analyses, we performed qPCR on all samples except 2 that did not have sufficient sample volume for the reactions. Only seven of the samples failed qPCR testing, meaning that for two or more housekeeping genes, their Ct values were >35 (see Supplemental Table S2 for the average Ct values of all housekeeping genes). Figure 1D–F shows linear correlation analysis plots of the Ct values for each housekeeping gene plotted against the other. Based on this analysis, we found strong linear relationships between each housekeeping pair, suggesting stable amplification in the clot RNA samples (R^2^ = 0.84 for *GPI* vs. *GAPDH*, R^2^ = 0.70 for *HPRT1* vs. *GPI*, and R^2^ = 0.64 for *GAPDH* vs. *HPRT1*).

### 3.4. RNA Sequencing Analysis on 48 Samples

**Figure 2:**
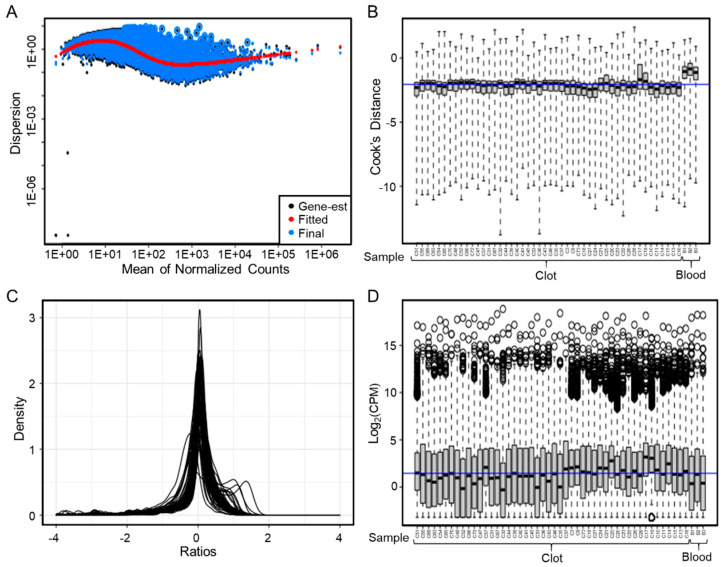
RNA Sequencing QC Analysis. (**A**) A dispersion plot in DESeq2 showing variation in the expression data. (**B**) A plot of Cook’s Distance in DESeq2 for each sample. (**C**) The distribution of the ratios in almost all cases was normal. (**D**) Boxplots of log-transformed CPM expression show no outlier samples. (Abbreviations: CPM = counts per million).

Select samples were subjected to RNA sequencing to test the feasibility in downstream transcriptomic applications. Libraries created using a poly-A pull-down kit, demonstrated good quality on an Agilent 2100 BioAnalyzer (Agilent, Las Vegas, NV) prior to sequencing. From the FastQC and MultiQC reports (Supplemental Table S3), the sequencing had an average of 35M reads (R1 and R2) across all clot samples with no poor-quality reads. The average percent GC and percent duplicate reads were 46% and 51%, respectively, which was similar to that of whole blood. On average clot, sequencing showed 18.3M assigned reads, with an average alignment rate of 95%, while whole blood sequencing showed a slightly greater (25.1M) assigned reads and average alignment rate (98%). (See Supplemental Figures S1–S4 for additional RNA sequencing quality data). As shown in Figure 2A, the dispersion plot in DESeq2 demonstrated variation within the expression data compared to whole blood. Furthermore, the calculation of Cook’s Distance in DESeq2 indicated no outliers in the samples selected for RNA sequencing (Figure 2B). The distribution of the ratios in almost all cases was normal (Figure 2C), indicating that the median ratio normalization (used by DESeq2) is appropriate for representing depth. Lastly, boxplots of log-transformed CPM expression showed no significant outliers, as all samples had similar median expression (around the blue line in Figure 2D).

### 3.5. Acellular Clots Yielded RNA of Lesser Quantity and Quality

**Figure 3:**
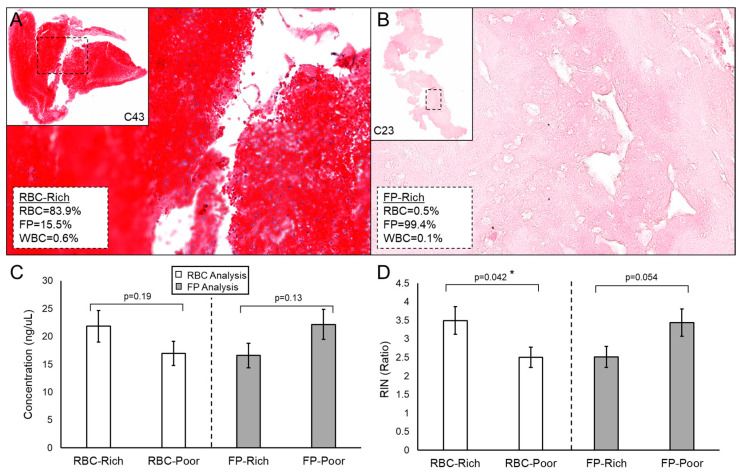
Histologic analysis of clots. (**A**) An example of an RBC-rich clot. (**B**) An example of an FP-rich clot that is largely acellular. (**C**) Average concentration in RBC-rich (≥50%) vs. RBC-poor (<50%) clots, and FP-rich (≥50%) vs. FP-poor (<50%) clots. (**D**) Average RIN in RBC-rich vs. RBC-poor clots and FP-rich vs. FP-poor. Error bars represent standard error, and “*” indicates statistical significance, *p* < 0.05 from Student’s *t*-test. (Abbreviations: FP = fibrin/platelet; RBC = red blood cell; RIN = RNA integrity number; WBC = white blood cell).

Clot RNA quantity and quality may be influenced by the composition of the clot, which may explain why some extractions failed or why some samples were unusable in downstream applications. We compared clot RNA concentration and RIN to their composition on histology in *n* = 52 of the cases. Figure 3A,B shows an example of RBC-rich (>50% RBC) and FP-rich (>50% FP) clots, respectively. In general, more cellular (RBC-rich) clots yield more RNA of significantly higher quality than acellular (FP-rich) clots (*p* = 0.042, Student’s *t*-test) (Figure 3C,D). There was no difference in concentration or RIN between WBC-rich (≥median WBC%) and WBC-poor (<median WBC%) clots (Supplemental Figure S5).

## 4. Discussion

The advent of thrombectomy therapy for large vessel AIS has enabled analyses of the retrieved clot material [13]. The biology of AIS clots could be related to clinically useful endpoints, such as stroke etiology, or could advance our knowledge into the pathology of the disease and biologic underpinnings of imaging biomarkers or treatment effectiveness. However, the molecular biology—specifically transcriptome signatures—of AIS blood clots, remains widely unknown. This may be due to difficulty in obtaining nucleic acid from clot tissue, which can be acellular or necrotic and not amenable to analysis with traditional RNA extraction methods. In this study, we developed a pipeline for extracting, quantitating, and analyzing gene expression profiles from AIS clots. To our knowledge, this work represents the first demonstration of successful RNA isolation followed by downstream qPCR and RNA sequencing applications for AIS clots.

Transcriptome profiling is a powerful tool for revealing expression patterns that are associated with complex diseases [9,15,16], such as stroke. The ability to extract satisfactory RNA from blood clots for downstream applications has been proven difficult with traditional extraction methods. Fraser et al. reported a collection method for AIS clots, in which the tissue was immediately flash frozen on dry ice [6]. Using a column-based RNA isolation kit (the RNeasy Mini) they extracted RNA from seven clot samples but failed to detect any usable RNA (no detectable RIN) and thus did not demonstrate any downstream applications. Baek et al. had more success in isolating AIS clot RNA, using RNALater for sample storage and phenol-chloroform-based methods (TRIzol) for RNA isolation [5]. In 82 cases, they performed qPCR analyses on clot mRNA for assessment of *IL-1β*, *IL-6, IL-8*, *IL-18*, *TNFα*, *MCP-1*, *MMP-2*, and *MMP-9*. However, they did not provide any RNA quantity and quality metrics and did not follow standard guidelines for qPCR (e.g., they used only one housekeeping gene and did not report primer efficiencies).

Other groups have reported successful isolation of quality RNA from other compromised vascular tissue samples. For example, Zakaria et al. used TRIzol and a column-based method (Qiagen QIAamp) to isolate RNA from frozen coagulated blood samples (average concentration = 26.3 ng/μL and average 260/280 = 1.71) and demonstrated qPCR analyses [17]. Additionally, Ahmed et al. used RNALater to store carotid atherosclerotic plaque samples and isolated RNA using Qiagen’s column-based RNeasy Fibrous Tissue Mini Kit (with proteinase K digestion) [18]. They reported average RNA concentrations of 27.8 ng/μL and RINs ranging from 0 to 8.6. Furthermore, they were able to fully detect *GAPDH* expression in all isolated RNA samples using qPCR.

Based on these previous reports, and our own unsuccessful attempts using column-based RNA extraction kits and phenol–chloroform-based methods, we chose to implement a magnetic bead-based method for isolating RNA that was combined with manual tissue disruption and proteinase K treatment. This protocol reduced human errors and low extraction efficiencies that can accompany phenol–chloroform and spin column-based techniques, because of the high binding affinity of the beads. This also aids in the isolation of nucleic acids from smaller tissues with low cellular composition. Based on our QC analyses, this method was able to extract RNA with comparable concentration, 260/280, and RIN values to that of Zakaria et al. [17] and Ahmed et al. [18]. However, the concentrations and RIN values of RNA from whole blood extracted on the same machinery were significantly greater. This may be due to the tissue properties of the clots, which can have varying cellular composition and phenotypes (e.g., some may be necrotic) [13]. Indeed, the histologic analysis showed clots that had lower cellular composition yielded RNA of lesser quantity and quality. One way we could have increased yields would have been to snap freeze the clots [19,20], but we did not implement this method due to the procedural complexity of keeping liquid nitrogen in surgical suites 24 h/day.

The primary goal of this work was to determine if the isolated RNA could be used in downstream applications. We found that the vast majority of RNA samples were viable for qPCR analysis using 10 ng of cDNA in 25 µL reactions. *GAPHD* expression was detectable in all samples, but *GPI* and *HPRT1* could not be detected in several samples, indicating a higher concentration of cDNA may be needed. Overall, linearity between Ct values among different housekeeping genes demonstrates stability in expression in the majority of RNA samples, indicating they can be used for downstream qPCR. On the other hand, fewer samples were amenable to RNA sequencing. Due to the fairly degraded nature of the samples, we opted to implement a library preparation assay to enrich mRNA using a poly-A pulldown. This may have been less ideal than rRNA depletion techniques (e.g., the Illumina TruSeq Stranded Total RNA Gold Kit), but preliminary tests (not shown) demonstrated better quality libraries using a poly-A selection. Despite this compromise, all libraries had greater than 20M reads that mapped to the human genome (or multiple genomes), and all but four had greater than 10M unique reads. Additionally, due to the amount of degradation in the samples, qPCR, which is more reliable (depends less on poly-A selection and only requires amplification of the target sequence), should be performed on significant genes identified by RNA sequencing as validation of expression.

This work has several limitations. First, we could not control the biologic composition or the size of the collected clot. These factors may influence the amount of starting material and the quality of the RNA we can extract. Second, due to procedural limitations, we stored retrieved clots in RNALater. Immediate storage in liquid nitrogen may better limit RNA degradation and yield higher quality extractions. Third, the RNA quality of many clot samples was low. While this does not pose a prohibitively significant problem for qPCR analysis, it may skew RNA sequencing results, particularly those sequencing of libraries created by poly-A selection. Therefore, in future studies, confirmatory qPCR of target genes identified via RNA sequencing will be necessary to validate differential expression. Lastly, we demonstrated success using a bead-based extraction method on the PerkinElmer Chemagic 360. This instrument and protocol are costly, which may limit widespread adoption of the procedure and could limit larger, future studies.

## 5. Conclusions

This is the first demonstration of the isolation and downstream application of RNA from AIS blood clots. Overall, nearly all of the 73 cases had enough RNA for qPCR analysis, while 48/73 were amenable for RNA sequencing. Analysis of both housekeeping gene amplification curves and standard RNA sequencing QC demonstrated the feasibility of downstream quantification applications for AIS RNA. Analysis of histology showed that acellular (FP dominant) clots tended to have lower RNA quantity and quality. These results lay the groundwork for future investigations into the molecular biology of AIS clots and their relationship to stroke pathophysiology, which are currently underway.

## Figures and Tables

**Table 1:** Description of patient population *.

Variable	Clot	Blood
Age (years, mean ± SE)	70 ± 1.7	73 ± 11
Sex		
Female	57%	67%
Co-Morbidities		
BMI (kg/m^2^, mean ± SE)	29 ± 1.0	28 ± 4.0
Smoking (%)	69%	67%
Hypertension (%)	80%	100%
Heart Disease (%)	29%	33%
Hyperlipidemia (%)	50%	33%
History of Cancer (%)	28%	33%
Diabetes (%)	29%	0.0%
Arthritis (%)	6.0%	33%
Asthma (%)	4.5%	0.0%
Clot location		
BA (%)	8.0%	0.0%
ICA (%)	28%	67%
MCA (%)	62%	33%
PCA (%)	2.0%	0.0%
Primary Treatment Method		
Stent Retriever (%)	10%	0.0%
Aspiration (%)	14%	0.0%
Solumbra (%)	76%	100%
tPA Administration		
IV-tPA (%)	0.0%	100%
IA-tPA (%)	36%	0.0%
Passes		
Number of Passes (mean ± SE)	1.9 ± 0.15	2.0 ± 1.0
Clot Retrieved on Pass (mean ± SE)	1.7 ± 0.15	2.0 ± 1.0
mTICI Score Post-Op.		
0 (%)	1.0%	0.0%
1 (%)	1.0%	0.0%
2a (%)	3.0%	0.0%
2b (%)	38%	0.0%
2c (%)	21%	67%
3 (%)	36%	33%
NIHSS		
NIHSS, Pre-Op. (mean ± SE)	16 ± 0.9	15 ± 5.0
NIHSS, After 24 h (mean ± SE)	10 ± 1.2	7.0 ± 5.0

* (Abbreviations: BA = basilar artery; BMI = body mass index; IA = intraarterial; ICA = internal carotid artery; IV = intravenous; MCA = middle cerebral artery; mTICI = modified thrombolysis in cerebral infarction; NIHSS = National Institutes of Health stroke score; PCA = posterior cerebral artery; SE = standard error; tPA = tissue plasminogen activator).

**Table 2:** RNA Quantity and Quality *.

Sample ID	Source	Conc. (ng/µL)	260/280	260/230	RIN
C1	Clot	<0.01	2.20	−0.31	NR
C2	Clot	3.4	1.97	0.33	3.8
C3	Clot	12.2	1.86	0.85	1.3
C4	Clot	<0.01	0.71	−0.71	NR
C5	Clot	1.6	2.46	0.34	2.7
C6	Clot	2.5	2.00	0.30	2.4
C7	Clot	6.5	1.87	0.56	1.6
C8	Clot	0.8	0.87	0.03	9.5
C9	Clot	10.3	1.93	0.84	1.4
C10	Clot	18.0	1.78	0.68	1.0
C11	Clot	17.1	1.97	0.95	2.5
C12	Clot	1.1	1.86	0.27	0.0
C13	Clot	2.3	6.14	0.20	2.6
C14	Clot	2.7	4.00	0.19	4.0
C15	Clot	7.8	2.69	0.4	2.4
C16	Clot	5.3	2.07	0.33	3.3
C17	Clot	10.6	1.77	0.29	2.8
C18	Clot	11.7	2.71	0.52	1.8
C19	Clot	46.0	1.71	1.14	2.7
C20	Clot	6.6	2.10	0.54	3.8
C21	Clot	4.6	2.22	0.31	2.4
C22	Clot	7.9	1.30	0.40	2.6
C23	Clot	15.4	1.15	0.37	0.0
C24	Clot	7.2	2.46	0.40	6.6
C25	Clot	<0.01	−0.79	0.04	1.4
C26	Clot	11.5	1.98	0.52	1.0
C27	Clot	7.5	2.65	0.40	1.0
C28	Clot	19.1	1.49	0.53	1.0
C29	Clot	29.1	1.89	0.58	2.3
C30	Clot	34.6	1.84	0.61	6.1
C31	Clot	40.2	1.84	0.59	2.4
C32	Clot	25.7	1.98	0.59	1.9
C33	Clot	34.4	1.82	0.55	2.0
C34	Clot	27.8	1.97	0.56	NR
C35	Clot	24.1	2.33	0.60	5.4
C36	Clot	29.8	1.95	0.56	6.1
C37	Clot	24.1	1.95	0.61	5.6
C38	Clot	33.4	1.87	0.61	2.3
C39	Clot	30.7	1.95	0.50	2.6
C40	Clot	23.6	1.98	0.49	4.4
C41	Clot	30.5	1.89	0.56	6.9
C42	Clot	5.2	2.78	0.85	2.5
C43	Clot	26.5	2.02	0.53	2.9
C44	Clot	30.0	2.04	0.54	4.9
C45	Clot	26.5	2.34	0.58	4.9
C46	Clot	35.6	2.11	0.63	3.7
C47	Clot	33.7	1.87	0.53	4.3
C48	Clot	31.9	1.89	0.54	3.2
C49	Clot	24.8	1.88	0.54	2.7
C50	Clot	39.3	1.94	0.58	NR
C51	Clot	13.8	1.83	0.91	2.1
C52	Clot	6.5	2.22	0.77	3.6
C53	Clot	27.9	1.86	0.5	1.6
C54	Clot	7.6	2.35	0.66	6.2
C55	Clot	30.7	2.01	0.56	NR
C56	Clot	8.1	6.47	0.65	2.6
C57	Clot	20.5	1.83	1.36	2.3
C58	Clot	<0.01	2.26	0.43	1.9
C59	Clot	<0.01	0.73	−1.13	4.8
C60	Clot	29.1	1.97	0.54	3.1
C61	Clot	35.7	1.88	0.58	NR
C62	Clot	13.1	2.34	0.61	NR
C63	Clot	31.8	2.01	0.61	NR
C64	Clot	30.8	2.03	0.60	NR
C65	Clot	23.3	1.93	0.58	NR
C66	Clot	28.0	1.94	0.55	NR
C67	Clot	29.2	2.08	0.54	4.3
C68	Clot	34.4	1.98	0.56	NR
C69	Clot	34.0	1.98	0.56	1.0
C70	Clot	23.1	2.34	0.62	1.0
C71	Clot	30.4	2.03	0.65	NR
C72	Clot	27.0	2.30	0.64	1.1
C73	Clot	30.8	1.85	0.53	7.1
B1	Blood	45.4	1.96	0.59	7.6
B2	Blood	55.1	1.87	0.75	4.8
B3	Blood	65.2	1.93	0.66	8.1

* (Abbreviations: Conc. = concentration; NR = not recorded; RIN = RNA integrity number).

## Data Availability

The data presented in this study are available upon reasonable request to the corresponding author. The data are not publicly available due to continued analysis by the corresponding author’s research team and IRB restrictions.

## Supplementary Materials

The following are available online at https://www.mdpi.com/article/10.3390/genes12101617/s1, Table S1: Primers used for qPCR, Table S2: Ct values for housekeeping genes from qPCR, Table S3: Sequencing quality metrics, Figure S1: FastQC Screen results, Figure S2: FastQC results, Figure S3: MultiQC read results, Figure S4: MultiQC mapping results, Figure S5: Histologic analysis of clots.
