# Supplementary material for: Isolation of RNA from Acute Ischemic Stroke Clots Retrieved by Mechanical Thrombectomy
Source: Genes (Basel). 2021 Oct 14;12(10):1617. doi: 10.3390/genes12101617 (PMC8536169; doi:10.3390/genes12101617)
Supplement: Supplementary file 1 [file genes-12-01617-s001.zip › genes-1413505-supplementary.pdf]

# Supplemental Data for: Isolation of RNA from Acute Ischemic Stroke Clots Retrieved by Mechanical Thrombectomy

**Vincent M. Tutino PhD[1,2,3,4], Sarah Fricano BS[1,2], Kirsten Frauens BS[1,3], Tatsat R. Patel MS[1,4], Andre Monteiro MD[1,3], Hamid H. Rai MD[1,3], Muhammad Waqas MBBS[1,3], Lee Chaves PhD[1,3], Kerry E. Poppenberg PhD[1,3] and Adnan H. Siddiqui MD-PhD[1,3,5]**

[1]Canon Stroke and Vascular Research Center; [2]Department of Pathology and Anatomical Sciences, [3]Department of Neurosurgery, [4]Department of Mechanical & Aerospace Engineering, [5]Department of Radiology, University at Buffalo, Buffalo, NY, USA

* Correspondence: **Author:** Vincent M. Tutino, PhD 875 Ellicott Street Canon Stroke and Vascular Research Center University at Buffalo Buffalo, NY 14203, USA. E-mail: vincentt@buffalo.edu; Phone: (716) 829-5400; Fax: (716) 854-1850

**Table S1.** Primers used for qPCR.*.

| *Transcript* | Primer Sequence | Avg. Annealing Temp. (°C) | Eff. | PCR Prod. Length (bp) |
|---|---|---|---|---|
| GAPDH *Housekeeping* | 5'-CGCTCTCTGCTCCTCCTGTT-3'<br>5'-CCATGGTGTCTGAGCGATGT-3' | 59.8 | 1.1 | 81 |
| GPI *Housekeeping* | 5'-AGGCTGCTGCCACATAAGGT-3'<br>5'-AGCGTCGTGAGAGGTCACTTG-3' | 61.7 | 0.95 | 240 |
| *HPRT1* *Housekeeping* | 5'-TGGCGTCGTGATTAGTGATG-3'<br>5'-CAAGACGTTCAGTCCTGTCC-3' | 57.4 | 0.82 | 131 |

*Primers were selected using Primer3 and NCBI's Primer Blast. (Abbreviations: Avg.=average, bp=base pair, Eff.=efficiency, Prod.=product, qPCR=quantitative polymerase chain reaction, Temp.=temperature).

**Table S2.** Ct values for housekeeping genes from qPCR.*.

| Sample ID | Sample Type | Ct (*GAPDH*) | Ct (*GPI*) | Ct (*HPRT1*) |
|---|---|---|---|---|
| C1 | Clot | 33.645 | NR | 37.800 |
| C2 | Clot | 25.420 | 28.625 | 30.400 |
| C3 | Clot | 24.795 | 28.220 | 30.550 |
| C4 | Clot | 28.915 | NR | 32.790 |
| C5 | Clot | 24.580 | 29.460 | 30.150 |
| C6 | Clot | 31.235 | 35.705 | 35.790 |
| C7 | Clot | 26.330 | 30.350 | 32.860 |
| C9 | Clot | 23.490 | 27.965 | 29.860 |
| C10 | Clot | 23.230 | 25.790 | 29.615 |
| C11 | Clot | 25.840 | 29.615 | 31.265 |
| C13 | Clot | 31.390 | 35.420 | 35.930 |
| C14 | Clot | 28.310 | 30.445 | 33.240 |
| C15 | Clot | 24.760 | 27.040 | 30.560 |
| C16 | Clot | 27.145 | 30.180 | 33.140 |
| C17 | Clot | 29.555 | 34.280 | 38.055 |
| C18 | Clot | 26.155 | 30.275 | 30.895 |
| C19 | Clot | 29.605 | 34.445 | 35.840 |
| C20 | Clot | 34.005 | NR | 36.260 |
| C21 | Clot | 28.130 | 32.375 | 35.630 |
| C22 | Clot | 27.535 | 30.415 | 32.210 |
| C23 | Clot | 27.920 | 30.115 | 32.340 |
| C24 | Clot | 28.840 | 30.535 | 34.360 |
| C25 | Clot | 26.920 | 29.765 | 34.235 |
| C26 | Clot | 25.575 | 28.650 | 31.555 |
| C27 | Clot | 25.270 | 29.080 | 31.725 |
| C28 | Clot | 24.335 | 27.590 | 30.980 |

| | | | | |
|---|---|---|---|---|
| C29 | Clot | 26.995 | 30.505 | 33.010 |
| C30 | Clot | 24.180 | 28.265 | 32.675 |
| C31 | Clot | 22.115 | 28.000 | 28.600 |
| C32 | Clot | 28.460 | 32.100 | 33.600 |
| C33 | Clot | 26.280 | 29.965 | 32.490 |
| C34 | Clot | 22.075 | 26.000 | 28.115 |
| C35 | Clot | 25.305 | 30.210 | 32.050 |
| C36 | Clot | 23.340 | 26.605 | 28.575 |
| C37 | Clot | 22.655 | 25.140 | 27.770 |
| C38 | Clot | 25.760 | 27.730 | 32.050 |
| C39 | Clot | 23.710 | 26.660 | 31.145 |
| C40 | Clot | 36.040 | 36.490 | 33.305 |
| C41 | Clot | 21.890 | 26.270 | 31.325 |
| C42 | Clot | 23.335 | NR | 31.670 |
| C43 | Clot | 26.825 | 31.180 | 31.900 |
| C44 | Clot | 23.680 | 27.245 | 30.080 |
| C45 | Clot | 21.415 | 25.910 | 30.040 |
| C46 | Clot | 25.435 | 27.895 | 32.915 |
| C47 | Clot | 24.820 | 29.555 | 32.810 |
| C48 | Clot | 26.075 | 30.400 | 33.690 |
| C49 | Clot | 27.680 | 31.710 | 34.785 |
| C50 | Clot | 25.290 | 28.285 | 32.825 |
| C51 | Clot | 26.345 | 30.860 | 32.875 |
| C52 | Clot | 25.240 | 28.890 | 32.625 |
| C53 | Clot | 22.950 | 26.500 | 30.580 |
| C54 | Clot | 29.915 | 33.745 | 32.010 |
| C55 | Clot | 22.915 | 26.900 | 29.895 |
| C56 | Clot | 24.155 | NR | NR |
| C57 | Clot | 22.900 | 27.315 | 30.595 |
| C58 | Clot | 28.145 | 34.230 | 35.630 |
| C59 | Clot | 24.995 | 28.970 | 31.490 |
| C60 | Clot | 29.270 | 33.090 | 36.295 |
| C61 | Clot | 21.830 | 27.625 | 29.775 |
| C62 | Clot | 22.090 | 27.735 | 31.885 |
| C63 | Clot | 29.525 | 32.110 | 34.815 |
| C64 | Clot | 23.995 | 26.815 | 28.830 |
| C65 | Clot | 30.98 | 39.780 | NR |
| C66 | Clot | 22.595 | 26.545 | 30.200 |
| C67 | Clot | 27.110 | 32.720 | 34.000 |
| C68 | Clot | 25.280 | 27.755 | 30.310 |
| C69 | Clot | 23.590 | 26.830 | 30.315 |
| C70 | Clot | 25.900 | 29.750 | 32.655 |
| C71 | Clot | 23.450 | 27.635 | 32.035 |
| C72 | Clot | 26.215 | 30.150 | 34.665 |
| C73 | Clot | 20.220 | 22.760 | 27.325 |
| B1 | Blood | 23.160 | 28.045 | 29.380 |
| B2 | Blood | 23.260 | 26.095 | 27.655 |
| B3 | Blood | 21.560 | 26.485 | 28.425 |

* (Abbreviations: NR=not recorded, qPCR=quantitative polymerase chain reaction).

**Table S3.** Sequencing quality metrics.*.

| | | FastQC | | | | | | | | MultiQC | | |
|---|---|---|---|---|---|---|---|---|---|---|---|---|
| **Sample ID** | **Sample Type** | **R1, Total Seq.** | **R1, No. Poor Qual. Seq.** | **R1, GC (%)** | **R1, Dup. (%)** | **R2, To-tal Seq.** | **R2, No. Poor Qual. Seq.** | **R2, GC (%)** | **R2, Dup. (%)** | **M Ass.** | **Ass. (%)** | **Alig. (%)** |
| C3 | Clot | 35.2 | 0 | 41 | 33.7 | 35.2 | 0 | 40 | 37.7 | 8.9 | 21.5 | 95.3 |
| C9 | Clot | 36.6 | 0 | 41 | 34.1 | 36.6 | 0 | 40 | 37.7 | 8.7 | 20.2 | 95.3 |
| C10 | Clot | 41.6 | 0 | 47 | 39.4 | 41.6 | 0 | 46 | 43.6 | 20.1 | 39.4 | 96.7 |

| | | | | | | | | | | | | |
|---|---|---|---|---|---|---|---|---|---|---|---|---|
| C11 | Clot | 34.3 | 0 | 45 | 35.9 | 34.3 | 0 | 44 | 41.0 | 14.3 | 34.0 | 96.3 |
| C13 | Clot | 22.4 | 0 | 47 | 55.6 | 22.4 | 0 | 46 | 61.8 | 10.8 | 37.3 | 92.5 |
| C14 | Clot | 29.5 | 0 | 43 | 49.5 | 29.5 | 0 | 42 | 54.2 | 6.0 | 16.2 | 91.9 |
| C15 | Clot | 33.6 | 0 | 46 | 41.6 | 33.6 | 0 | 45 | 47.2 | 16.0 | 38.3 | 95.8 |
| C16 | Clot | 20.3 | 0 | 45 | 54.1 | 20.3 | 0 | 44 | 59.8 | 7.9 | 29.9 | 92.3 |
| C17 | Clot | 33.8 | 0 | 41 | 27.6 | 33.8 | 0 | 37 | 30.2 | 2.0 | 4.7 | 94.3 |
| C18 | Clot | 41.0 | 0 | 45 | 42.4 | 41.0 | 0 | 45 | 47.7 | 20.3 | 40.2 | 96.5 |
| C19 | Clot | 41.8 | 0 | 46 | 37.3 | 41.8 | 0 | 44 | 41.9 | 6.2 | 11.0 | 94.9 |
| C20 | Clot | 30.9 | 0 | 46 | 48.0 | 30.9 | 0 | 46 | 54.7 | 11.2 | 26.9 | 95.8 |
| C21 | Clot | 31.2 | 0 | 43 | 51.5 | 31.2 | 0 | 41 | 54.5 | 6.8 | 18.2 | 93.1 |
| C22 | Clot | 26.9 | 0 | 47 | 48.7 | 26.9 | 0 | 46 | 55.4 | 15.1 | 44.9 | 96.5 |
| C23 | Clot | 32.1 | 0 | 44 | 50.7 | 32.1 | 0 | 42 | 56.2 | 11.1 | 27.6 | 94.6 |
| C24 | Clot | 29.2 | 0 | 44 | 44.0 | 29.2 | 0 | 43 | 50.1 | 10.2 | 28.0 | 95.5 |
| C25 | Clot | 34.1 | 0 | 41 | 30.7 | 34.1 | 0 | 40 | 33.2 | 4.4 | 10.7 | 94.8 |
| C26 | Clot | 34.0 | 0 | 41 | 36.3 | 34.0 | 0 | 42 | 40.7 | 10.0 | 24.9 | 95.4 |
| C27 | Clot | 42.4 | 0 | 49 | 51.5 | 42.4 | 0 | 48 | 56.3 | 14.0 | 17.1 | 97.0 |
| C28 | Clot | 40.6 | 0 | 47 | 44.8 | 40.6 | 0 | 46 | 49.2 | 19.5 | 37.7 | 96.5 |
| C31 | Clot | 37.7 | 0 | 46 | 55.1 | 37.7 | 0 | 47 | 62.9 | 27.0 | 61.4 | 97.0 |
| C32 | Clot | 20.5 | 0 | 45 | 74.5 | 20.5 | 0 | 44 | 81.2 | 14.2 | 56.0 | 91.0 |
| C33 | Clot | 31.3 | 0 | 46 | 65.8 | 31.3 | 0 | 46 | 73.6 | 23.3 | 63.3 | 96.2 |
| C35 | Clot | 36.1 | 0 | 48 | 65.1 | 36.1 | 0 | 47 | 72.3 | 29.0 | 63.4 | 97.2 |
| C36 | Clot | 70.8 | 0 | 47 | 52.8 | 70.8 | 0 | 47 | 58.9 | 46.2 | 52.3 | 97.1 |
| C37 | Clot | 41.8 | 0 | 47 | 49.5 | 41.8 | 0 | 48 | 55.8 | 28.8 | 54.2 | 97.5 |
| C39 | Clot | 33.9 | 0 | 47 | 55.3 | 33.9 | 0 | 47 | 63.2 | 23.8 | 56.8 | 96.9 |
| C40 | Clot | 47.9 | 0 | 49 | 61.5 | 47.9 | 0 | 49 | 68.0 | 33.2 | 49.5 | 95.7 |
| C41 | Clot | 43.5 | 0 | 49 | 48.7 | 43.5 | 0 | 49 | 54.3 | 30.9 | 58.9 | 97.5 |
| C43 | Clot | 27.3 | 0 | 47 | 61.5 | 27.3 | 0 | 46 | 70.5 | 19.1 | 54.0 | 95.3 |
| C44 | Clot | 41.5 | 0 | 50 | 56.4 | 41.5 | 0 | 50 | 63.4 | 29.2 | 53.1 | 97.3 |
| C45 | Clot | 41.2 | 0 | 50 | 58.2 | 41.2 | 0 | 50 | 65.4 | 29.5 | 52.8 | 97.4 |
| C46 | Clot | 30.3 | 0 | 48 | 56.3 | 30.3 | 0 | 48 | 64.7 | 21.0 | 52.9 | 95.5 |
| C47 | Clot | 27.9 | 0 | 49 | 60.8 | 27.9 | 0 | 49 | 69.2 | 18.9 | 51.5 | 92.3 |
| C48 | Clot | 20.7 | 0 | 48 | 66.3 | 20.7 | 0 | 47 | 73.7 | 12.9 | 44.2 | 89.4 |
| C51 | Clot | 36.3 | 0 | 44 | 40.5 | 36.3 | 0 | 42 | 45.9 | 11.9 | 25.5 | 95.2 |
| C52 | Clot | 30.1 | 0 | 46 | 63.4 | 30.1 | 0 | 46 | 72.3 | 23.0 | 63.0 | 96.0 |
| C54 | Clot | 40.8 | 0 | 49 | 58.1 | 40.8 | 0 | 49 | 65.4 | 22.6 | 30.4 | 97.0 |
| C55 | Clot | 35.0 | 0 | 47 | 55.2 | 35.0 | 0 | 47 | 63.2 | 25.6 | 58.1 | 97.0 |
| C57 | Clot | 31.5 | 0 | 42 | 29.0 | 31.5 | 0 | 40 | 32.7 | 7.1 | 18.7 | 94.8 |
| C63 | Clot | 42.7 | 0 | 48 | 52.2 | 42.7 | 0 | 48 | 58.4 | 30.4 | 58.3 | 97.3 |
| C65 | Clot | 42.4 | 0 | 50 | 59.5 | 42.4 | 0 | 50 | 65.1 | 22.9 | 29.1 | 97.4 |
| C66 | Clot | 20.3 | 0 | 48 | 66.6 | 20.3 | 0 | 48 | 74.1 | 13.7 | 57.8 | 89.3 |
| C67 | Clot | 39.4 | 0 | 47 | 60.3 | 39.4 | 0 | 47 | 68.3 | 30.4 | 66.4 | 97.5 |
| C69 | Clot | 27.0 | 0 | 46 | 56.6 | 27.0 | 0 | 47 | 61.4 | 17.6 | 51.7 | 96.0 |
| C70 | Clot | 28.6 | 0 | 46 | 50.2 | 28.6 | 0 | 47 | 58.0 | 15.1 | 43.3 | 95.6 |
| C72 | Clot | 34.7 | 0 | 46 | 59.3 | 34.7 | 0 | 47 | 66.7 | 20.7 | 48.4 | 95.2 |
| C73 | Clot | 42.8 | 0 | 46 | 49.4 | 42.8 | 0 | 46 | 55.4 | 27.9 | 52.1 | 97.1 |
| B1 | Blood | 39.5 | 0 | 53 | 66.7 | 39.5 | 0 | 54 | 71.1 | 21.6 | 35.3 | 97.8 |
| B2 | Blood | 37.6 | 0 | 55 | 65.0 | 37.6 | 0 | 54 | 76.0 | 27.2 | 40.3 | 98.2 |
| B3 | Blood | 42.4 | 0 | 54 | 69.4 | 42.4 | 0 | 54 | 72.7 | 26.4 | 45.3 | 97.7 |

* (Abbreviations: Alig.=aligned, Ass.=assigned, Dup.=duplicate, M=million, No.=number, Qual.=quality,R1=strand 1, R2=strand 2, Seq.=sequences).

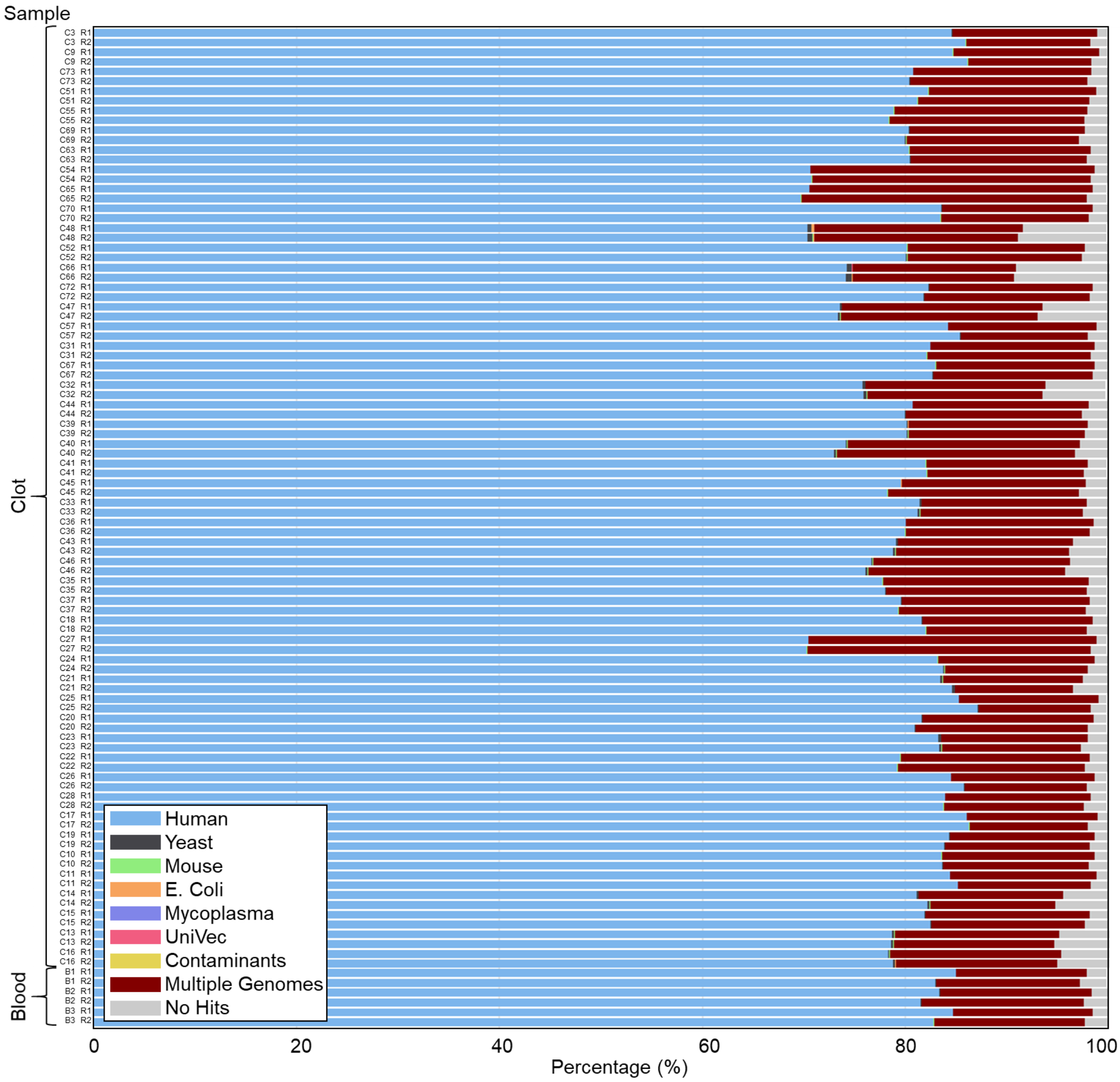


**Figure S1. FastQC Screen results.** Percentages of reads show mapping predominantly to the human genome or multiple genomes.

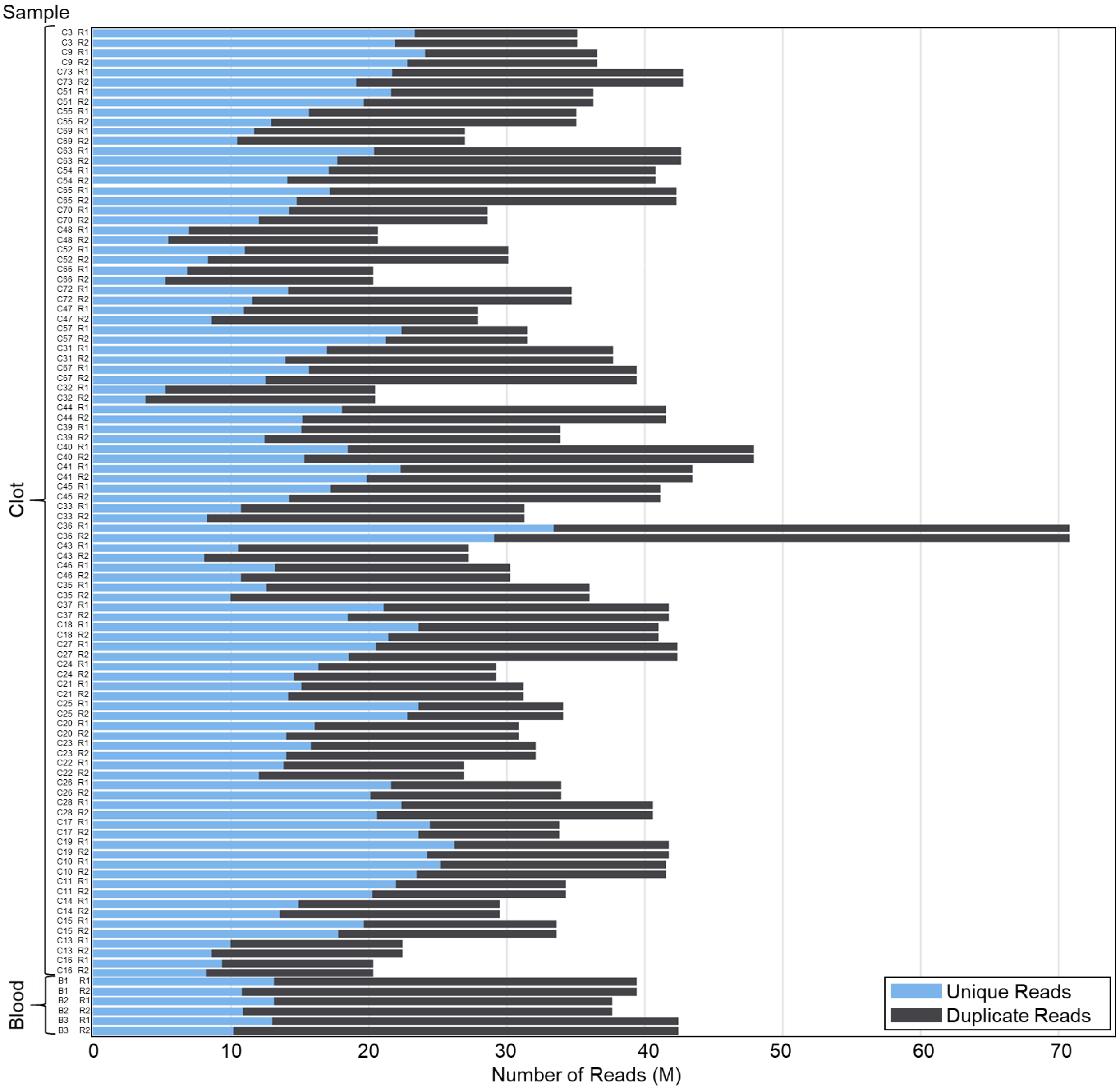


**Figure S2. FastQC results.** Number of unique and duplicate reads for each transcriptome.

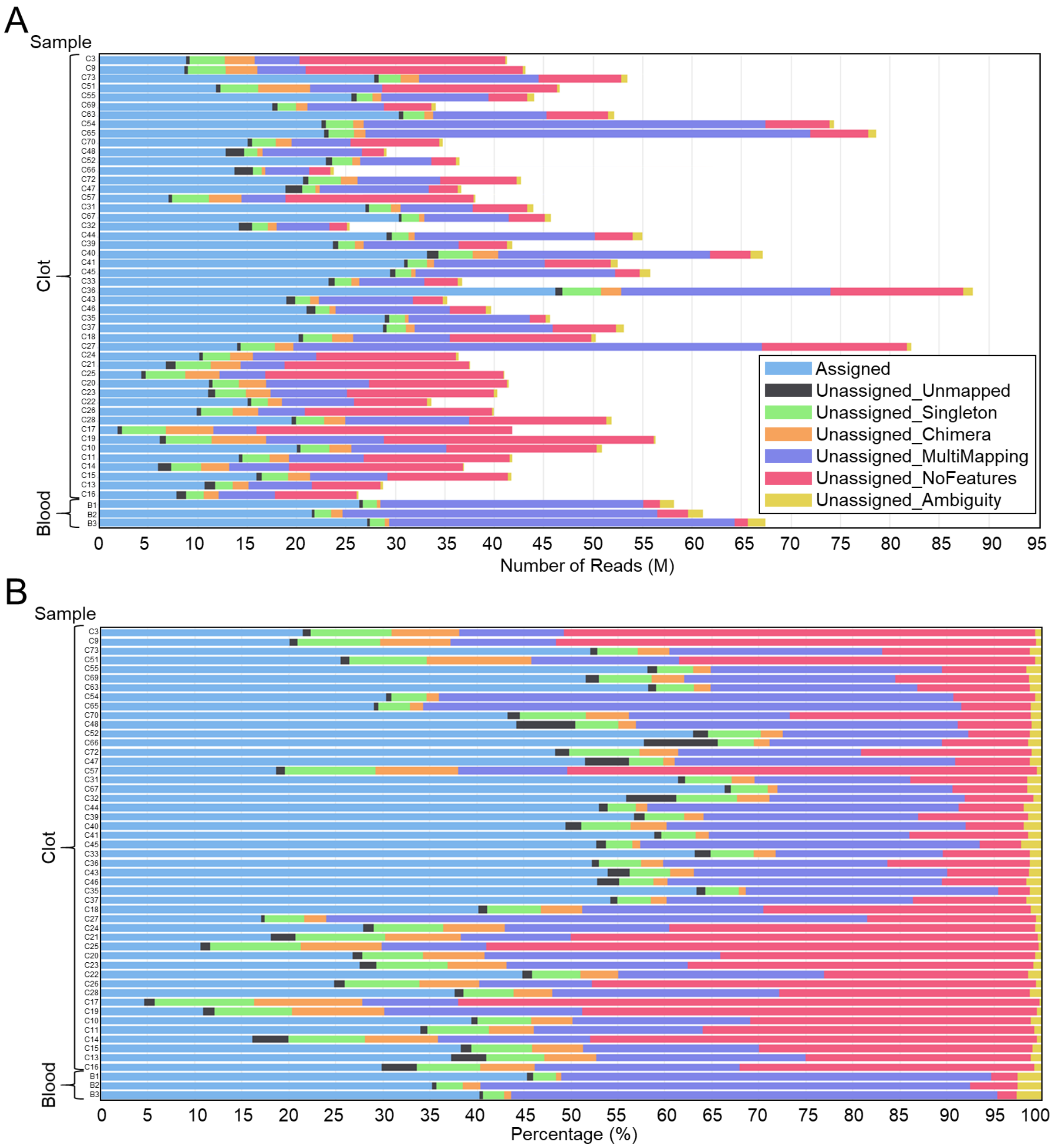


**Figure S3. MultiQC read results.** The A). number and B). percentage of reads for each transcriptome.

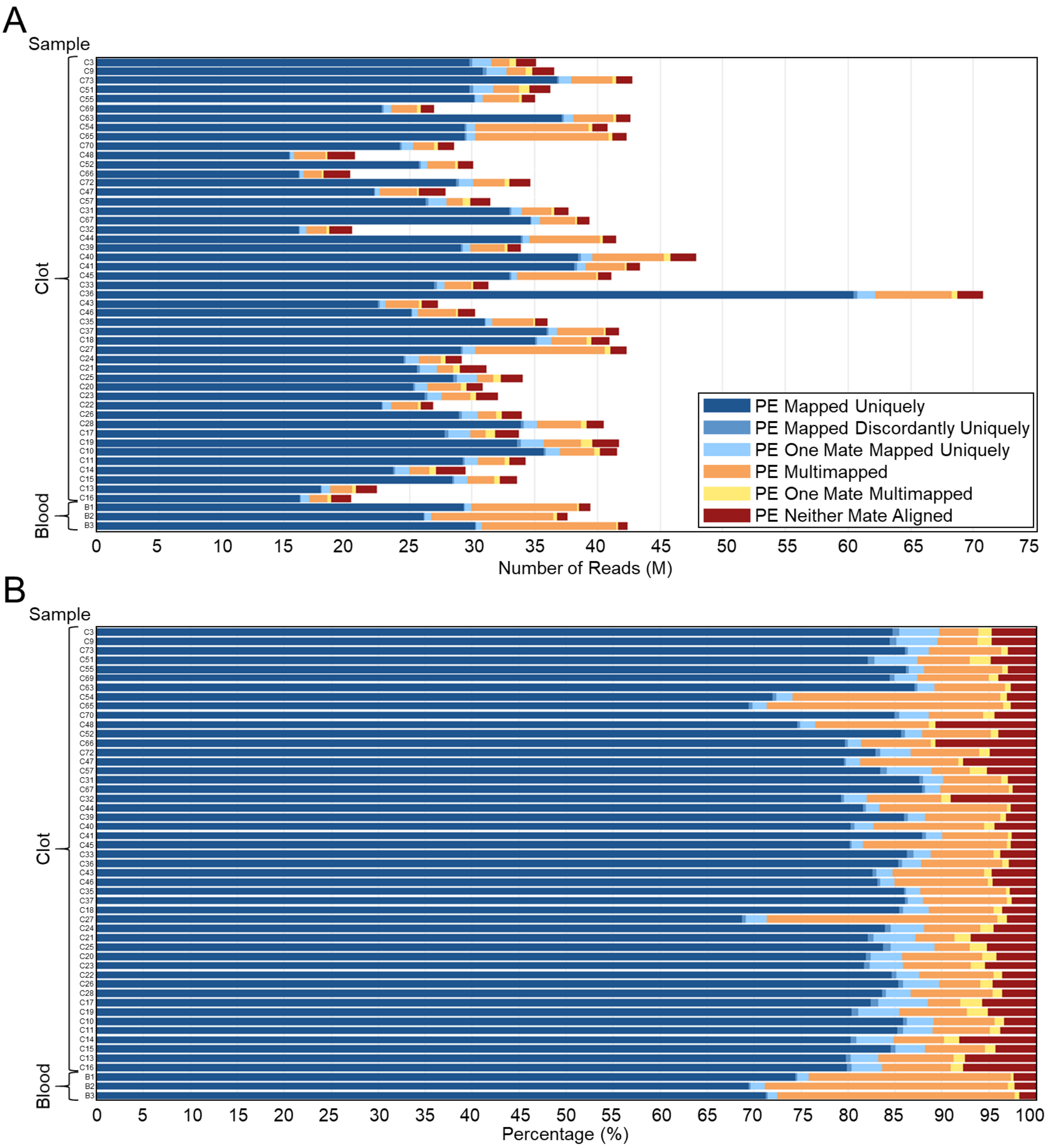


**Figure S4. MultiQC mapping results.** The **A).** number and **B).** percentage of read mapping for each transcriptome.

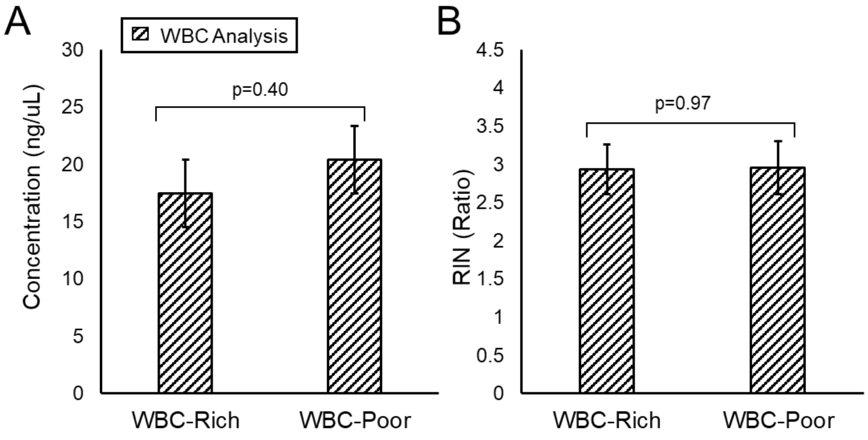


**Figure S5. Histologic analysis of clots. A).** Average concentration in WBC-rich (≥median WBC %) vs. WBC-poor (<median WBC %) clots. **B).** Average RIN in WBC-rich vs. WBC-poor clots. Values are averages, and error bars represent standard error. (Abbreviations: RIN=RNA integrity number, WBC=white blood cell).
